# Benefits to Plant Health and Productivity From Enhancing Plant Microbial Symbionts

**DOI:** 10.3389/fpls.2020.610065

**Published:** 2021-04-12

**Authors:** Gary Harman, Ram Khadka, Febri Doni, Norman Uphoff

**Affiliations:** ^1^Department of Plant Pathology, Cornell University, Geneva, NY, United States; ^2^Department of Plant Pathology, The Ohio State University, Columbus, OH, United States; ^3^Nepal Agricultural Research Council, Directorate of Agricultural Research, Banke, Nepal; ^4^Institute of Biological Sciences, University of Malaya, Kuala Lumpur, Malaysia; ^5^CALS International Agriculture Programs, Cornell University, Ithaca, NY, United States

**Keywords:** holobiont, endophyte, plant, bacteria, fungi

## Abstract

Plants exist in close association with uncountable numbers of microorganisms around, on, and within them. Some of these endophytically colonize plant roots. The colonization of roots by certain symbiotic strains of plant-associated bacteria and fungi results in these plants performing better than plants whose roots are colonized by only the wild populations of microbes. We consider here crop plants whose roots are inhabited by introduced organisms, referring to them as Enhanced Plant Holobionts (EPHs). EPHs frequently exhibit resistance to specific plant diseases and pests (biotic stresses); resistance to abiotic stresses such as drought, cold, salinity, and flooding; enhanced nutrient acquisition and nutrient use efficiency; increased photosynthetic capability; and enhanced ability to maintain efficient internal cellular functioning. The microbes described here generate effects in part through their production of Symbiont-Associated Molecular Patterns (SAMPs) that interact with receptors in plant cell membranes. Such interaction results in the transduction of systemic signals that cause plant-wide changes in the plants’ gene expression and physiology. EPH effects arise not only from plant-microbe interactions, but also from microbe-microbe interactions like competition, mycoparasitism, and antibiotic production. When root and shoot growth are enhanced as a consequence of these root endophytes, this increases the yield from EPH plants. An additional benefit from growing larger root systems and having greater photosynthetic capability is greater sequestration of atmospheric CO_2_. This is transferred to roots where sequestered C, through exudation or root decomposition, becomes part of the total soil carbon, which reduces global warming potential in the atmosphere. Forming EPHs requires selection and introduction of appropriate strains of microorganisms, with EPH performance affected also by the delivery and management practices.

## Introduction

Plants, like other so-called higher organisms, do not exist as entities unto themselves. They are biotic systems which consist of the plant plus innumerable microorganisms, the plant microbiome. This review considers plant-associated bacteria and fungi, focusing on those that internally colonize plant roots as microbial endophytes. Plants, together with their associated microbiomes, function as complex multi-species entities, referred to in the literature as holobionts (Margulis and Fester, 1991). The association can be detrimental to the plant if pathogens predominate, or it can be neutral. More often, it results in plants having better health, growth, and performance. We have previously discussed endophytic root colonization and the resulting symbiotic increases in plants’ capabilities using the concept of Enhanced Plant Holobionts (EPHs; Harman and Uphoff, 2019).

A number of mechanisms are involved in producing these effects, particularly plant-microbial interactions, but there are also various microbe-microbe interactions. Because they are not widely known, we are particularly interested here in the microbial production of organic molecules that interact with plant cell membranes and induce system-wide changes in plant physiology, altering both plant gene and protein expression. These molecules we refer to as Symbiont-Associated Molecular Patterns (SAMPs; Harman and Uphoff, 2019). This terminology is consistent with the scientific literature on plant pathogens that already uses the terms Pathogen-Associated Molecular Patterns (PAMPs) and Damage-Associated Molecular Patterns (DAMPs); see, for example, Malik et al. (2020). As a consequence of certain plant-microbial interactions, plants’ photosynthesis can be enhanced, and there can be induced improvements in internal cellular environments. A result of these effects is having significantly larger EPH roots and shoots with higher crop yields.

By enhancing yields, EPHs can contribute to maintaining food security and reducing hunger in the world. These goals will become more challenging in future decades as still-rising human populations must be supported from a diminishing natural resource base that will be further constrained by the changing climate. EPHs can help to mitigate this as increased plant photosynthesis with greater root growth can increase carbon sequestration from the atmosphere and larger root systems will increase carbon stores in the soil. Increases in soil carbon (SC), especially in soil organic matter (SOM), will improve soil health and fertility which are associated with greater plant health, crop yields, and ultimately human health.

Following this introduction, Section 2 (entitled the functioning of enhanced plant holobionts) describes in general terms the physical interactions of symbiotic microbes with plants, and especially the colonization of internal plant organs and the nature of the endophytic associations. Section 3 (mechanisms for enhanced plant holobionts) reviews mechanisms by which endophytes react with plants to protect plants’ health and support their growth, robustness, and productivity. Section 4 (biochemical and genetic effects associated with EPHs) discusses the biochemical effects associated with SAMPs and their gene and protein regulation, improvements in photosynthetic efficiency, and effects of internal cellular functioning.

Section 5 (benefits conferred on plants) considers benefits that this symbiotic association confers on plants, including the control of biotic stresses, including disease, insect pests, and nematodes, as well as the mitigation of abiotic stresses such as drought, salt, and adverse temperatures. Section 6 (agricultural and societal benefits) discusses higher-level agricultural and societal benefits, including improvements in soil health, enhancing sustainable food production, and creating environmental benefits such as carbon sequestration and storage, which can help to slow global warming by capturing and removing greenhouse gases such as CO_2_ from the atmosphere.

Section 7 (management and delivery systems for EPHs) addresses important aspects of the delivery and management of microbial agents, including the application of exogenous inoculum and the mobilization of existing soil populations. Section 8 (enhancing microbial endowments with changes in crop and soil management) then goes into management systems such as no-till cultivation that can have beneficial effects on the soil biota, and the system of rice intensification for which we have experimental evidence confirming the practicality of EPHs when modifying crop management. Section 9 summarizes the various components that contribute to the creation and cultivation of EPHs and to the benefits that these confer at both micro and macro levels. Section 9 is a summary of the paper.

Numerous fungi and bacteria may provide beneficial effects including endophytic fungi (*T. afroharzianum, viride, atroviride, virens, reesei* and others; Woo et al., 2014; Harman and Uphoff, 2019; Ikram et al., 2019), in addition to other endophytic fungi such as strains of *Aspergillus niger* (Ismail et al., 2020), *Penicillium roqueforti* (Ikram et al., 2018), *Aspergillus terreus* (Khushdil et al., 2019) *Yarriwia lipolytica* (Farzana et al., 2019), as well as *Piriformaspora indica* (Gill et al., 2016) *Penicillium citrinum* and *Aspergillus terreus* (Waqas et al., 2015), and bacteria such as *Pseudomonas* (Pieterse et al., 2014), *Bacillus* (Kloepper et al., 2004) and Rhizobiacae (Chi et al., 2005). It is important to note that, while many fungi and bacteria are endophytic and provide benefits to plants, microbial effects on plants are highly specific to individual strains. Just because one strain benefits certain plants, does not mean that all members of the same genus or species of microbes will have the same effects.

## The Functioning of Enhanced Plant Holobionts

### Microbial Enhancement of Plants

Many bacteria and fungi are beneficial to plants in various ways:

- By improving their resistance to diseases and pests;
- By mitigating abiotic stresses such as drought, salt, and adverse temperatures;
- By improving plants’ nutritional status through better acquisition of nutrients from the soil, enhancing supply of nutrients such as through the fixation of nitrogen, and better nutrient-use efficiency;
- By enhancement of plants’ photosynthetic capability; and
- By maintaining internal cellular environments that are more conducive for the functioning of critical plant metabolic processes.

These services generally result in better growth of plants’ shoots and roots and thus in higher yields, especially under adverse conditions.

Numerous bacteria and fungi are known to improve plant performance, among them bacteria in the genus or families *Pseudomonas, Bacillus*, and Rhizobiacae, and fungi such as *Trichoderma* and *Piriforma*. Other microbes may also induce similar benefits, but the groups mentioned here are the best documented, most studied, and most widely used. For a comprehensive listing of beneficial microbes, see (Copping, 2004). The most effective strains of either fungi or bacteria are usually ones that colonize the plant roots endophytically.

### Endophytic Root, Leaf, and Branch Colonization

Many of the most beneficial microbes live within the internal space of plant roots. In at least one case, they take up residence anywhere in the plant where they are applied, not limited to the roots. In two other cases, once established in the roots, the microbes become systemic throughout the plant. The beneficial species that are considered in this review do not cause disease or other deleterious effects. Not all, but many microorganisms living within plant organs and tissues have beneficial effects on plants’ growth, health, and productivity. Their symbiotic services to plants are similar to those that myriad microbes in the human microbiome confer on our species. These endophytes when introduced purposefully to augment whatever microbial populations exist naturally in the plants’ microbiomes create what we refer to as EPHs.

**Figure 1:**
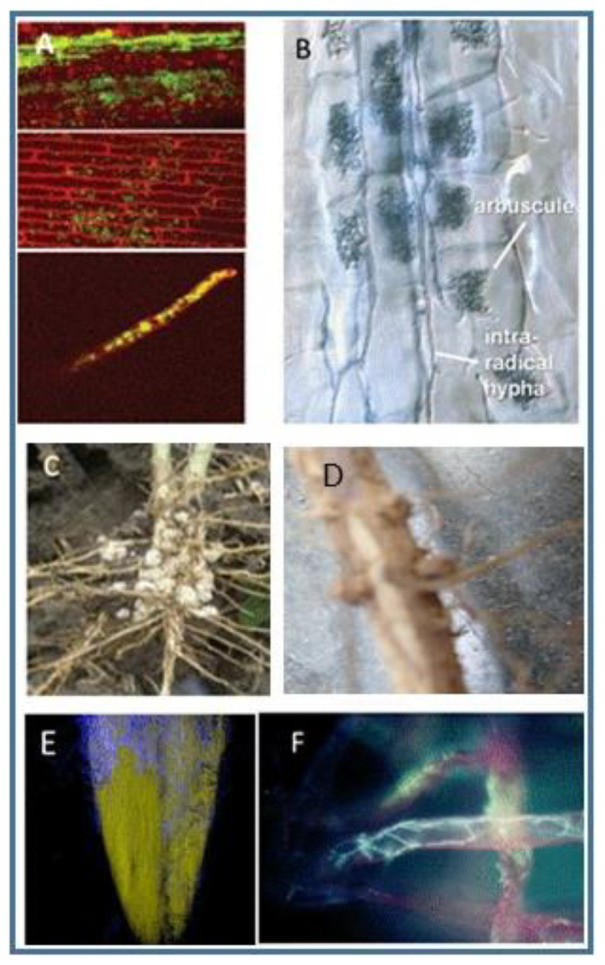
Examples of endophytic colonization by different bacteria or fungi. **(A)** Endophytic colonization of rice by *Rhizobium leguminosarum bv. Trifoli* in rice. The photomicrograph shows the bacteria in the stems after the bacterium was applied to seeds. From (Chi et al., 2005). The journal does not require persmission to use materials. **(B)** Photograph of mycorhizzae labeled to show arbucules and hyphae within roots, from (Schussler, 2009), used with permission of the author. **(C)** Nodules on soybean roots. Used courtesy of Advanced Biological Marketing. **(D)** Photograph of nodules on soybean roots. Photo by the first author. **(E)** A photomicrograph of fluorescent imaging of a corn root showing growth of *Trichoderma* on the surface. Spores of the fungus were added to seeds, and the growth shown was taken 2 days later. Photograph courtesy of Advanced Biological Marketing. **(F)** Fluorescent photomicrograph of *Trichoderma* growing in root hairs. From (Harman et al., 2004a), used with permission of the journal.

Colonization patterns differ as shown in Figure 1. As noted above, the beneficial effects are always strain-specific, and no generalizations can be made regarding specific taxa since a given strain or species may have members that are highly beneficial, while other members of the same species may not confer advantages. Some genera contain species whose colonization confers advantages to the plants that harbor them, while other genera, e.g., *Pseudomonas, Fusarium*, and *Rhizoctonia*, include both pathogens and beneficial organisms. Some strains of *Pseudomonas* can be beneficial symbionts for certain plants, while other strains cause disease in both animals and plants.

Among the more complex interactions are those involving bacteria in the Rhizobiacae family of protobacteria. In leguminous plant species, these bacteria infect plant roots and form nitrogen-fixing nodules, structures composed of both plant and bacterial cells as shown in Figures 1C,D.

Conversely, some of these same bacteria which fix nitrogen in the roots of clover plants can infect the roots of cereal plans like wheat or rice and become systemic throughout these plants (Figure 1A; Chi et al., 2005). Little is known about the extent to which such systemic colonization occurs in other plants.

Vascular-arbuscular mycorrhizal (VAM) fungi also form complex plant root-microbial structures. These fungi interact with plant roots to form arbuscules within the roots that are highly efficient in transferring nutrients such as phosphorus from the VAM to the plant and of nutrients from the plant to the VAM in return, as seen in Figure 1B.

Some strains of *Trichoderma* become endophytes and colonize the cortical cells of roots. Figures 1E,F show this interaction. If specific strains of *Trichoderma* are applied to seeds, spores of the fungi germinate very rapidly, and within a few days hyphae, branching filaments, grow from the seed onto the emerging radicle (Figure 1E). The fungi then colonize the roots (Figure 1F). Other strains of *Trichoderma*, however, are unable to colonize the rhizosphere or to become endophytic (Harman and Uphoff, 2019).

In certain plants, strains of *Trichoderma* become systemic, living throughout the plant, while in other plants these same strains are confined to the roots (Bae et al., 2011). The location of endophytes is therefore plant-specific. Other fungi may become endophytic in any part of the plant where they are inoculated. For example, *Clonostachys rosea* can colonize plant tissues including leaves, with the result of controlling certain plant diseases that affect the leaves (Shafia et al., 2001; Xue et al., 2014).

Chemical crosstalk between roots and root colonizing microbes is required for symbiotic relationships to occur. With maize, roots and *A. nominus* require indole acetic acid and flavonoids to establish the relationship (Mehmood et al., 2020). Similar crosstalk occurs to establish the relationship with legumes and Rhizobiacae and with mycorrhizae and the numerous plants they colonize. These signaling pathways developed first with mycorrhizae and the legume-Rhizobiacae developed similar interactions (Genre and Russo, 2016). Other relationships, such as those between *Fusarium* and legumes employ elements of these same pathways (Skiada et al., 2020).

## Mechanisms for Enhanced Plant Holobionts

### Mycoparasitism

The ability of certain fungi to colonize and parasitize other fungi and Oomycetes has been known for almost 90 years. This capability of fungi does not per se lead to the formation of holobionts, but it is included here for reasons of completeness. In addition, some fungi that are mycoparasiitic are endophytes that form holobiontic associations with plants.

The events that occur in mycoparasitism have been well documented in interactions of *Trichoderma* with other fungi, including pathogens. The interactions can be complex. At least *in vitro*, *Trichoderma* can be grown in a directed fashion to attack targeted fungi (Chet et al., 1981). This is facilitated by the ability of the mycoparasite to secrete small amounts of an exochitinase that releases carbohydrates; *Trichoderma* sensing these follows this trail of nutrients to the target fungi (Brunner et al., 2003). Once *Trichoderma* comes into contact with the target fungus, it engages in interaction that results, in some cases, with the coiling response noted above.

Once in contact with the target fungus, *Trichoderma* can penetrate its internal lumen and establishes an internal infection (Harman et al., 2004a). This is facilitated by the ability of *Trichoderma* to produce a potent array of fungal cell-wall-degrading enzymes (Figure 1D) and also various antibiotics, many of which exhibit synergistic fungitoxicity. Scores of genes, enzymes, and metabolites are known to be involved in this interaction (Harman et al., 2004a; Lorito et al., 2010). In some cases, mycoparasitism is essential and sufficient for biocontrol.

For example, strains of *Ampelomyces* are parasitic on powdery mildews (Kiss et al., 2004), and since they are obligate pathogens on mildews, this mechanism must be the operative one. *Coniothyrium minitans* is a commercial product for control of sclerotia-producing fungal pathogens. *C. minitans* parasitizes the sclerotia which contain food reserves for the pathogen, and over time this reduces the pathogenic populations, resulting in successful control (Whipps and Lumsden, 1991; Whipps, 1997; Gwynn, 2014).

### Production of Antibiotics

Numerous metabolites are produced by bacteria and fungi that are toxic to particular plant pathogens and pests. For example, certain strains of *Agrobacterium* that do not give rise to galls in plants have the ability to control other strains of the bacteria that do. This effect is associated with the microbe’s production of agrocin, a biochemical that is highly toxic to tumorigenic bacterial strains (Burr and Reid, 1994). It cannot be ascertained whether this toxin is solely responsible for the observed biocontrol ability, however, since other factors such as competition for infection sites could be operative.

Similarly, control of the take-all disease in wheat caused by *Guamannomyces tritici* can be controlled by the addition of strains of *Pseudomonas fluorscens* that produce phenazine antibiotics (Fravel, 1988; Weller, 1988). This bacterium was obtained from soils that became suppressive to the disease following years of wheat monoculture. This microorganism can create a soil in which the disease is reduced to levels where crop damage is greatly reduced (Cook, 2017).

In another case, we searched for strains of *Trichoderma* that could control the *Phytophthora* disease caused by pathogenic Oomycetes. Hundreds of strains were screened, first *in vitro*, and then by plant assays. The only strain identified with an ability to control this pathogen was a strain of *T. virens* that produced the antibiotic gliotoxin (Smith et al., 1990). This antibiotic is extremely toxic to zoospores of the pathogen (Wilcox et al., 1992).

This strain was isolated from a soil that had been periodically cultivated with peas for over a century. The root rot caused by the water mold *Aphanomyces euteiches* f. sp. *pisi* was not present in this soil, although earlier reports indicated that in the pathogen had caused very damaging root rot in this same area. The pathogen *A. euteiches* is a relative to the plant-damaging Oomycete *Phytophthora* and both produce motile zoospores, so the gliotoxin produced by *T. virens* is extremely toxic to both.

In the cases mentioned above, the antibiotics are clearly associated with disease suppression. However, it should not be assumed that this antibiotic production is the only mechanism involved in disease control. Many or most microorganisms produce at least some antibiotic substances. While their antibiotics may be involved in biocontrol, in most cases other mechanisms may also be involved, as discussed in later sections.

### Antifungal Enzymes

Earlier, we mentioned fungitoxic enzymes from *Trichoderma* that are involved in mycoparasitism. In addition, various antibiotics that are produced have antifungal synergy with certain enzymes. The diversity of these enzymes and their functions is remarkable. There are multiple classes of enzymes that degrade chitin, cellulose, proteins, and other polymers found in nature. Beyond this, there are numerous separate enzymes for each functional classification, and the numbers of active enzymes and combinations are very large.

For example, a search for *Trichoderma*-produced chitinases in the Uniprot database gave 1,090 entries (https://www.uniprot.org/uniprot/?query=trichoderma+chitinase&sort=score–chitinases are one of several classes of cellulose-degrading enzymes), while a similar search for the broader category of *Trichoderma* cellulases gave 9,559. Some of these are from different strains that produce similar enzymes, but a perusal of this database demonstrates that many are different enzymes produced by beneficial microorganisms, having, e.g., dissimilar molecular weights.

### Competition

For many years, this has been considered one of the three main mechanisms for microbial disease control, along with antibiosis and mycoparasitism. It is obvious that competition occurs widely and can never be excluded as a mechanism of biocontrol, but it is hard to prove or disprove. In at least one case, the control of aflatoxin by heavy application of atoxigenic strains of *Aspergillus flavus*, competition is clearly the main factor. The atoxigenic strains compete with the one that produces aflatoxin, and this prevents accumulation of levels of this highly-toxic metabolite that are harmful to animals, including humans (Garber and Cotty, 1997).

Competition for infection sites by nonharmful strains can be expected to exclude harmful strains or species that are deleterious. It is thus difficult to rule competition in or out, since many cases of biocontrol may include competition.

### Siderophores

Siderophores are a class of microbial metabolites that sequester iron, binding to it very tightly and making it difficult for other microbes with a lower affinity for iron to compete with those that are producing siderophores with a very strong binding of iron (Trapet et al., 2016). Because iron under many soil conditions is poorly soluble, plants and other organisms in soil have evolved mechanisms to acquire iron under these conditions, either through the production of siderophores or through acidification or the immediate area in proximity to the organism. Organisms that produce siderophores with a very high affinity to iron can compete successfully with other organisms (Trapet et al., 2016).

Siderophores from fluorescent *Pseudomonas* strains (e.g., pyoverdines and pseudobactins) are among the strongest iron-binding compounds known. They can solubilize iron in the soil and to transport it back into the microorganism, which enables it to obtain this vital compound for its metabolism. In so doing, it scavenges iron away from other microbes, and this is an important mechanism in the competitive battle with other organisms that would occupy this niche.

This capability can be demonstrated through several methods, such as comparing the Fe-EDTA or EDDHA (Scher and Baker, 1982; Hubbard et al., 1983). These compounds chelate iron and make it available to plants and microorganisms. If the synthetic compound reverses the biological effect, this is evidence of the role of the siderophore in that effect. This approach was taken in both of the citations just given. The effect may be either beneficial or detrimental. The strong effect of the *Pseudomonas* siderophore has been shown to be important in biocontrol of *Fusarium* wilt (Scher and Baker, 1982).

However, competition for iron prevented biocontrol of damping-off diseases by a strain of *Trichoderma* in some New York soils that had low levels of available iron; however, it was effective in Colorado, where Fe carbonates were more available. In the NY soils, biocontrol could be accomplished in the presence of synthetic iron-chelating compounds. A search for *Trichoderma* strains in NY soils that could be effective in biocontroil resulted in the identification and selection of a strain that provided biocontrol and which was found to produce siderophores of its own (Hubbard et al., 1983). These two examples demonstrate the importance of this mechanism.

### Induced Systemic Resistance

The previous sections have described biocontrol by the action of one organism on another. However, it is becoming increasingly clear that a great deal of the control of diseases and pests, as well as control of abiotic stresses, is a result of the effects of the microbial agent on the plant, changing gene and protein expression that results in benefits from developing EPHs.

We noted above, for example, that *T. virens* produces the antibiotic gliotoxin. For many years this antibiotic was assumed to be responsible for the *Trichoderma’s* ability to control seedling diseases of cotton (Howell, 2003). However, we now know that antibiotic production was not the primary mechanism of control in this case. Studies in which mutants of *T. virens* were produced have shown varying capacities for biocontrol, mycoparasitism, and antibiotic production. The only property correlated with the ability of *T. virens* to control *R. solani*, which causes sheath blight in rice, for example, was the *Trichoderma’s* ability to induce plant resistance to the pathogen, as there was no correlation of biocontrol with either antibiotic production or mycoparasitism (Howell et al., 2000; Harman et al., 2004a).

**Figure 2:**
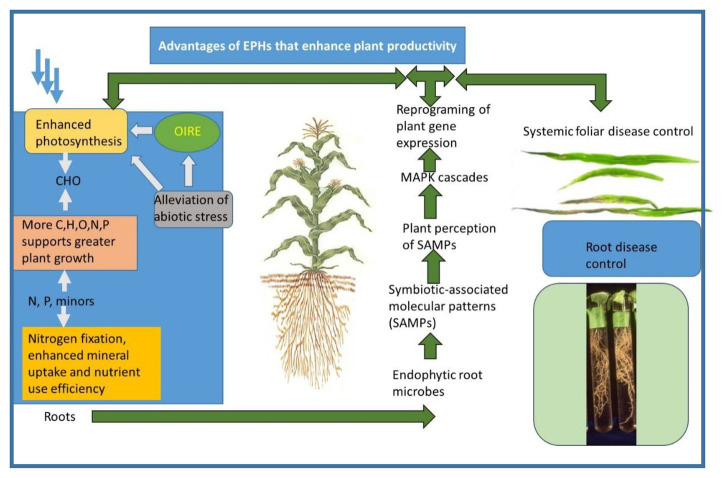
A synoptic diagram of the interactions of plants and endophytes that result in the formation of EPHs. A similar diagram was presented in (Harman and Uphoff, 2019), which is an open access journal. The photograph of corn leaves is from (Harman et al., 2004a) and is used with permission of the journal.

Evidence of induced resistance includes the ability to control abiotic or biotic stresses at a distance from where the agent is located (Harman et al., 2004a). For example, many organisms are restricted to plant roots, but induce plant resistance to foliar diseases. An example of shown in Figure 2 in the photograph of corn leaves, where *T. afroharzianum* was either applied or not applied to cpheorn seeds. The fungus is restricted to growth only in the roots, but it induces plant resistance to anthracnose on its leaves. In the photo, the two upper leaves were from a plant whose seeds had been treated, while on the lower leaves, no treatment was applied. Clearly, much less disease occurred on leaves from treated plants, thus demonstrating the concept of protection at plant sites distant from the actual location of the fungus.

Other evidence comes from genetic studies. For example, the gene NPR1 is essential to functioning of both Systemic Acquired Resistance (SAR) and Induced Systemic Resistance (ISR). These are two separate biochemical pathways by which each kind of resistance is induced. In studies with strains of the plant *Arabidopsis* that were deficient in the gene encoding this factor, *Trichoderma* did not control the fungal pathogen *Pythium* while strains with functional NPR1 were well-protected by the fungus (Shoresh et al., 2010).

However, these strains are also potent inducers of systemic resistance in plants (Pieterse et al., 2014), so attribution of causation is not unambiguous.

These two examples (many others could be cited) demonstrate that effects on plants of beneficial microbes occur as a consequence of microbial effects on plants, rather than just their effects on plant pathogens as provided in section 5. Most of the studies demonstrating this effect began to be published in the early 2000s cf. (Harman et al., 2004a). Older literature frequently ascribed the benefits of microbial agents to other mechanisms, but this was based on imcomplete knowledge. The ability of endophytic microbes to directly affect plants is the primary element contributing to the superiority of EPHs. To understand this, we turn to biochemistry.

## Biochemical and Genetic Effects Associated with EPHs

### Production and Description of SAMPs

In almost all cases, the induction of systemic effects which protect plants from pathogenic losses is mediated by the production of signaling molecules by endophytic microbes that interact with plant cell membranes. This results in signal transduction that occurs, in many cases, through mitogen-activated protein (MAP) kinases (Shoresh et al., 2010). Two principal pathways for effects on plants are the ISR and SAR mentioned above. ISR uses jasmonic acid as a principal signaling molecule, while SAR relies on salicylic acid. There is a considerable amount of cross-talk between these two pathways, and the interactions and triggering systems have been provided diagrammatically elsewhere (Harman et al., 2004a; Pieterse et al., 2014).

As indicated in those and other references, wounding of plants by injury or insects, infection by pathogenic microorganisms, and especially the effects of endophytic beneficial organisms all result in induction of beneficial systemic effects as does the alleviation of the negative effects of abiotic stresses.

The signaling compounds produced by beneficial organisms are numerous and varied. The primary elicitors for Rhizobiace and AMFs are chitooligosaccarides, and these are part of the common symbiotic pathway (Gough and Cullimore, 2011; Genre and Russo, 2016). The elicitor in *P. indica* is cellotriose (Johnson et al., 2018). *Trichoderma* uses a number of different compounds that trigger systemic activity including sulfur-rich proteins including SM1 (Djonovic et al., 2007) and certain hydrophobins (Ruoccco et al., 2015). These proteins contain a hydrophobic and a hydrophilic portion that enables them to insert themselves into cell membranes. These elicited responses frequently act by signal transduction (Shoresh et al., 2006).

Surfactins from *Bacillus subtlis* also have hydrophobic and hydrophilic portions of the molecule, and they too induce systemic reactions in plants. Both can be characterized by Ca^++^ bursts in the plant cells to which they are applied (Jourdan et al., 2009). There may be small-molecular-weight volatile compounds such as 1-octen-3-ol (Harman et al., 2018) and 6-pentyl-*α*-prone (Lorito et al., 2010). Other non-volatile small-molecular-weight compounds such as koningic acid, also known as heptelidic acid, act as elicitors of systemic plant responses (Lorito et al., 2010) is required for the systemic response of *T. virens* (Djonovic et al., 2007). Any of the small molecular compounds used alone can elicit systemic responses in plants (Harman and Uphoff, 2019).

### Interaction of SAMPs With Cellular Membranes

Signal transduction leads to systemic responses in plants (Ward and Schroeder, 1977; Shoresh et al., 2006). The amphoteric molecules (those with both hydrophobic and hydophilic regions) such as the proteins and surfactins just mentioned interact with cellular membranes through the production of pores in the membranes that open channels for ion influx and efflux and also trigger Ca^++^ bursts and H_2_O_2_ production that are characteristic of resistance responses.

This in turn leads to signal transduction that leads to systemic responses (Shoresh et al., 2010). This transduction is indispensable for the systemic response that EPHs induce, eliciting resistance to biotic and abiotic stresses. Signal transduction is induced by the interactions of SAMPs produced by the microorganism, as described earlier. This in turn leads to signal transduction which leads to systemic responses (Shoresh et al., 2010). This transduction is indispensable for the systemic response that EPHs induce, eliciting resistance to biotic and abiotic stresses. Signal transduction is induced by the interactions of SAMPs produced by the microorganism, as described earlier (Harman and Uphoff, 2019). In addition to the SAMPs described in that reference, plant hormones such as salicylic acid and jasmonic acid can act a elicitors of plant responses, In addition, the methylated forms can also be involved in systemic transport throughout the plant (Morita et al., 2009; Shah and Zeier, 2013).

### Signal Transduction and Systemic Changes in Plant Genes and Proteins

As mentioned above, systemic changes in plant gene and protein expression require signal transduction, at least in some cases, through the action of MAP kinases (Shoresh et al., 2006). This results in changes in plant genes and proteins, frequently as up-regulation. There are many thousands of proteins and genes that are known to be up-regulated. For example, a study of microbial inoculation of rice identified 2,414 transcripts that were differentially expressed in plants whose roots had been endophytically colonized by *Trichoderma*, thereby affecting plant growth and health (Doni et al., 2019a; Harman et al., 2019). These differently-expressed genes included genes directly involved in photosynthesis (335 transcripts), in the synthesis of photosynthetic products, and in the protection and maintenance of cellular functions (Doni et al., 2019a; Harman et al., 2019). Other examples are provided in section 8.

An important consideration in this up-regulation are the energy costs to the plant. The pathways involved require both energy and resources such as fixed carbon, nitrogen, and other components that also are necessary for plant growth and development (Shoresh and Harman, 2008). As part of meeting the needs for both plant growth and effective responses to stresses, and to economize on their expenditure of resources, plants employ a process called gene priming. In this process, a stimulus such as either stress or a response to plant endophytes induces an initial response in the plant which then establishes processes whereby proteins and other elements can be produced more rapidly and in larger quantity in the future. A plant that is primed by previous experience or by induced resistance can make responses that are quicker and stronger than would otherwise occur (Conrath et al., 2015).

This process may take the form of some modification of histones surrounding DNA or DNA methylation which are part of the plant’s regulatory machinery (Jaskiewicz et al., 2011a,b). These changes may be long-lasting and may even persist in the next generation. They are an efficient mechanism to minimize the cells’ energy costs while still permitting rapid responses by the plant to stressors or other demands, while preserving energy and resources for plant growth when these are not required by environmental or other internal requirements (see section 5). This reprogramming of gene expression is shown pictorially (Figure 2).

## Benefits Conferred on Plants

### Control of Biotic Stresses Including Diseases, Insect Pests, and Nematodes

Conventional agriculture practices use chemical biocides in controlling plant diseases and pests. Unfortunately, this approach induces resistance in pathogens and pests during long-term use (Mahmood et al., 2016). The excessive use of biocides in agriculture can also cause undesirable effects on humans and the natural environment (Heong et al., 2015). The use of EPHs is an underutilized opportunity for sustaining high agricultural production with lower negative impacts (Harman and Uphoff, 2019). Several endophytes belonging to different genera are involved in eliciting induced systemic resistance in plants and reported to be an effective tool for the biological control of certain plant pathogens and pests (Compant et al., 2019; Doni et al., 2019a).

The development of high-throughput molecular techniques such as proteomics, genomics, metagenomics and metabolomics for microbe isolation and characterization have allowed for the identification of a variety of bacteria and fungi able to act beneficially when proliferating in the soil for disease control and plant-growth promotion. Multi-omics has also advanced our ability to strategically select for microorganisms that have unique host site-specific qualities that function in biological disease or pest control (Bonanomi et al., 2018).

*P. roqueforti* and *T. reesei* produced numerous substances that had broad-spectrum activity against several bacterial plant pathogens, as well as antioxidative compounds (Ikram et al., 2019). *P. citrinum* and *A. terrus* are endophytes that secrete gibberelins were evaluated for the ability to protect against stem rot caused by *Scleerotium rolfsii*. These endophytes reduced disease. The authors concluded that the endophytes reprogramed the plants involved in systemic plant defense reactions (Waqas et al., 2015).

Application of microbes as biocontrol agents in the field can have many benefits without requiring extensive investment in plant breeding and genetics (Harman et al., 2019). These beneficial microbes can provide benefits through various mechanisms such as competing with pathogens or by directly antagonizing plant pathogens by producing certain antibacterial or antifungal compounds (Ab Rahman et al., 2018); induction of plant genes and biochemical pathways that provide greater resistance to disease and pest through induced systemic resistance (Harman et al., 2019); modulation of the signaling and metabolism of reactive oxygen species (ROS) as a key mechanism to counteract microbial antagonism (Nath et al., 2016); changing microbial balance in the rhizosphere in favor of beneficial microorganisms, thus suppressing a broad spectrum of diseases and pests (Siddiqui, 2005).

The *Trichoderma* spp. controls plant diseases through various mechanisms, e.g., mycoparasitism and production of antibiotics. However, more recent studies have indicated that a primary method of pathogen control occurs through the ability of the *Trichoderma* spp. to reprogram plants’ gene expressions so as to lead to the induction of systemic resistance (Shoresh et al., 2010; Poveda et al., 2019). For example, *T. asperellum* has been able to control white rot disease caused by *Sclerotium cepivorum* in onion plants by up-regulating the expression of biocontrol-related genes such as *AcPR1*, *AcPAL1*, *AcLOX1*, and *AcEIN3* (Rivera-Mendez et al., 2020).

The ability of *T. asperellum* to control white rot is only a subset of its capabilities. They can control disease, and have other benefits, including enhancement of growth, yield, and physiological parameters (Rivera-Mendez et al., 2020). Moreover, a study in inoculated rice seedlings revealed that *T. asperellum* can up-regulate numerous genes that were involved in plant defense responses and in systemic acquired resistance (SAR) signaling (Doni et al., 2019a). An abundance of up-regulated genes that relate to defense response and SAR signaling is an indication of greater plant ability to resist biotic stresses.

Some endophytic fungi, particularly *Beauveria bassiana*, have demonstrated antagonistic activity against numerous insect pests and arthropods (Mascarin and Jaronski, 2016). A recent study has indicated that *B. bassiana* could endophytically colonize pecan seedlings by seed soaking, seed coating, and soil drenching. The establishment of endophytic *B. bassiana* in pecan plants led to a protection against two pecan aphids in particular, namely *Melanocallis caryaefoliae* and *Monellia caryella* (Ramakuwela et al., 2020).

Moreover, the presence of *B. bassiana* in maize plants has reduced the survival and fecundity of the aphid *Sitobion avenae*, an important pest in agricultural systems (Mahmood et al., 2019). It has been suggested that protective alkaloids developed by the plant in response to endophytic fungal colonization could play an important role in protecting plants against chewing insects (Gange et al., 2019). In addition to producing protective alkaloids, endophytes promote plant immunity against chewing insects by enhancing endogenous defense responses mediated by the jasmonic acid (JA) pathway (Bastias et al., 2017).

Arbuscular mycorrhizal fungi (AMF), which are endosymbionts, are effective biocontrol agents against several soil-borne plant pathogens. AMF are obligate root symbionts that can offer many benefits and protect their host plant against pathogen infections (Schouteden et al., 2015). For example, the biocontrol efficacy of AMF against common bean root rot caused by *Fusarium solani* f. sp. *Phaseoli* has showed that plants grown with symbiotic AMF exhibited greater resistance against the pathogen compared to untreated plants (Eke et al., 2016).

The results also revealed that total soluble phenols, flavonoids contents, and the defense enzyme phenylalanine ammonia lyase (PAL) were increased with AMF colonization, indicating an enhancement of the plant immune system against *F. solani* f. sp. *Phaseoli* (Eke et al., 2016). A transcriptomic analysis of soybean plantlets in symbiosis with AMF versus non-AMF soybean plantlets following infection by *F. virguliforme* showed a comprehensive view of changes in gene expression, confirming an alteration of gene-expression patterns by AMF. There were 1,768 differentially expressed genes (DEGs) in the AMF-colonized plants compared to 967 DEGs in the noncolonized plants. Major transcriptional changes corresponded to defense response-related genes belonging to secondary metabolism, stress, and signaling categories (Marquez et al., 2019).

Finally, several plant growth-promoting bacteria (PGPR) also possess traits that make them well suited as biocontrol and growth-promoting agents. As a consequence, plants treated with PGPR may be larger and healthier and have greater yields than plants without such treatment (Mhatre et al., 2019). *Pseudomonas* and *Bacillus* species are two PGPR species that are capable of protecting *Solanum lycopersicum* against bacterial wilt disease. These PGPRs produced biocatalysts for plant-growth promotion and regulated pathogen-related genes such as *PR-la* and *PAL* (Durairaj et al., 2018).

Canola plants inoculated with *P. chlororaphis* showed greater protection against the necrotrophic fungus *Sclerotinia sclerotiorum*. The *P. chlororaphis* treatment resulted in structural and metabolic changes, and it prevented the accumulation of ROS in the leaf of canola plants. Global transcriptional analysis showed a significant up-regulation of genes associated with SAR mechanisms, ROS signaling, and chloroplast integrity (Duke et al., 2017). In addition, *P. chlororaphis* was capable of suppressing *S. sclerotiorum* by secreting the antibiotics pyrrolnitrin and phenazine, degradative enzymes, and siderophores (Nandi et al., 2017).

Microorganisms have developed mechanisms that increase their host fitness and provide a more stable ecological community by working effectively against a broad spectrum of pathogens and pests and for alleviation of the adverse effects of plant stress, which is discussed in the next section.

### Alleviation of Abiotic Stresses

On a daily basis, plants are exposed to numerous harmful environmental conditions that significantly affect their growth (Shahid et al., 2018). EPHs are reported to have substantial impacts on plants by enabling plants to grow consistently faster and more uniformly whether the stress was drought, salt, cold or flooding (Cheng et al., 2019). Over the years, some EPHs, especially fungal endophytes, have been widely regarded to be an important tool that can mitigate plant stress through various cellular plant processes (Harman, 2011; Rodriguez et al., 2019).

#### Drought Stress

Drought stress, one of the most destructive abiotic stresses, has increased in intensity over the past decades, endangering the world’s food security. Currently, 90% of global water consumption is used for agriculture, and water constraints are expected to intensify in the future (Huang et al., 2019). Numerous microbes can confer fitness benefits to plants, including increased tolerance to drought stress (Rodriguez and Redman, 2005).

There are various agronomic mechanisms that explain microbes’ contributions to reducing the impacts of drought: alterations in root architecture that result in improved water use efficiency; increases in the synthesis of osmolytes, particularly proline; increases in antioxidant enzymes that scavenge for ROS; manipulation of phytohormones; and modifying plant gene regulation (Ngumbi and Kloepper, 2016).

Root colonization by *Pseudomonas chlororaphis* induces systemic drought tolerance in *Arabidopsis thaliana* by increasing transcript accumulation from genes associated with defense, response to ROS, and phytohormone-responsiveness, accompanied by decreases in transcription factors associated with ethylene and abscisic acid signaling (Cho et al., 2013). *P. simiae* promoted growth and protected soybean plants from damage from drought stress, mainly manifested as changes in the gene expression profiles of drought stress-responsive genes such as *DREB2A*, *EREB*, *GOLS* and *P5CS*, and secondarily as modulation of phytohormonal signaling altered plant physiology and morphology (Vaishnav and Choudhary, 2019).

Inoculation of wheat with *Trichoderma* enhances drought tolerance through induced changes in stomatal conductance, net photosynthesis, and chlorophyll fluorescence. The inoculation also decreases proline, malondialdehyde (MDA), and hydrogen peroxide (H_2_O_2_), while increasing total phenolics and plant capacity to scavenge ROS (Shukla et al., 2015). *Trichoderma* inoculation in rice plants results in significant increases in plant height, total dry matter and chlorophyll content (Doni et al., 2014). Other studies have demonstrated the ability of *Trichoderma* to improve drought tolerance in rice by modulating proline, superoxide dismutase, and lipid peroxidation products and altering the transcript level of dehydrins-related genes such as *LOC_Os01g50700*, *LOC_Os02g44870*, and *LOC_Os11g26750* (Shukla et al., 2015). Further, *Trichoderma* has improved the ability of tomato plants to neutralize damaging ROS when exposed to a water deficit. The ability of tomato plants to protect themselves from oxidative damage was accompanied by the up-regulation of genes encoding antioxidant enzymes that significantly reduce the glutathione and ascorbate contents (Mastouri et al., 2012).

The application of *Alternaria*, a fungal endophyte, conferred drought tolerance on tomato plants. Endophyte-colonized plants exposed to drought had higher root and shoot biomass, better water-use efficiency, and higher photosynthetic efficiency, and they also recorded lower ROS than non-colonized plants (Azad and Kaminskyj, 2016). Moreover, application of *Curvularia protuberata* has been found to reduce rice plants’ water consumption by 20–30% and increase their growth rate, reproductive yield, and biomass when exposed to drought stress (Redman et al., 2011). These different findings indicate that symbiotic endophytes can be useful in mitigating the impacts of drought stress on plants, which will unfortunately increase with climate change.

#### Salt Stress

Salinity is another major abiotic stress that limits the growth and productivity of plants in arid and semiarid environments (Thu et al., 2017). To cope with salt stress, plants deploy a variety of traits to control cell functioning and development that rely on signal perception, signal integration, and processing plant systems (Hanin et al., 2016). ROS are overproduced in plants when they are exposed to abiotic stresses such as salinity. However, we must bear in mind that despite their toxicity, ROS are also regulators of growth, development, and defense pathways, so their elimination is not desirable (Farooq et al., 2019). Balancing the generation and reduction of ROS is a crucial process in plants (Rodriguez and Redman, 2005).

Many studies have reported microbes’ abilities to induce salt stress in plants by modulating ROS production (Harman and Uphoff, 2019). Inoculation of chickpea plants with *Bacillus subtilis* under saline conditions increased plant biomass and photosynthetic pigments, while reducing the levels of ROS and lipid peroxidation. *B. subtilis* has also been found to decrease Na accumulation while enhancing the N, K, Ca, and Mg content in the plants (Abdallah et al., 2018). *B. amyloliquefaciens* inoculation of rice plants under salt stress increased rice plant growth and the expression of several stress related genes such as *NADP-Me2, EREBP, SOSI, BADH, SERK1, GIG*, and *SAPK4* (Nautiyal et al., 2013).

In another study, barley plants previously inoculated with *Piriformospora indica* have been shown to increase resistance of salt stress. *P. indica* appeared to confer tolerance to this stress, at least partly, through the up-regulation of ascorbate and antioxidant enzymes (Baltruschat et al., 2008). Similarly, colonization of tomato plants by *P. indica* increased the expression of an important gene in leaves, namely LeNHX1, one of a family of genes responsible for removing sodium from cells. Further, *P. indica* also increased levels of antioxidant enzyme activity, offering further protection (Abdelaziz et al., 2019).

*Enterobacter* spp. have improved vegetative growth and alleviated salt stress in tomato plants by producing 1-aminocy-clopropane-1-carboxylate (ACC) deaminase and indole-3-acetic acid (IAA) which resulted in increased fresh weight, dry weight, and plant height. At the molecular level, treatment with *Enterobacter* spp. has increased the expression of salt-stress-related genes such as *P5CS1*, *P5CS2*, *MPK3* and *MPK6* (Kim et al., 2014). *Dietzia natronolimnaea* *STR1* protected wheat plants from salt stress by stimulating the expression of *TaST* (a salt stress-induced gene), and *SOS1* and *SOS4* (salt-sensitive-pathway-related genes), accompanied with enhanced gene expression of various antioxidant enzymes such as *APX*, *MnSOD*, *CAT*, *POD*, *GPX*, and *GR* (Bharti et al., 2016).

*Y. lipolytica* minimized the effects of salt on corn growth and development through the plant hormones absicic acid and indole acetic acid, indole-3-acetmimide and flavonoids. Treated plants had higher levels of chlorophyll, carotenes, and the antioxidants perioxidase and catalase (Farzana et al., 2019).

#### Cold and Heat Stress

Cold stress also severely hampers the reproductive development of plants and will result in significant agricultural losses. The major negative effect of cold stress is that it induces severe membrane damage in plants (Yadav et al., 2010). To improve cold stress in plants, cold-tolerant microorganisms such as *Trichoderma* spp., *Burkholderia* spp., and *Pseudomonas* spp. are usually used (Mishra et al., 2011). Inoculation of *B. phytofirmans* increased grapevine growth and starch, proline, and phenolic contents at a low temperature (Barka et al., 2006). With wheat plants, bacterization with *Pseudomonas* spp. under cold-induced stress significantly increased growth, total chlorophyll, anthocyanin, total phenolics, and starch content, and decreased the Na^+^/K^+^ ratio, and electrolyte leakage compared to non-bacterized control (Mishra et al., 2009).

*Pseudomonas* spp. were also reported to mitigate impacts of cold stress in tomato plants by reducing membrane damage and ROS levels, improved antioxidant activity in leaf tissues, and altering expression of cold acclimation genes *LeCBF1* and *LeCBF3* (Subramanian et al., 2015). Furthermore, endophytic *T. harzianum* increased the tolerance of tomato plants under chilling stress by reducing the lipid peroxidation rate and electrolyte leakage while increasing leaf water content and proline accumulation. *T. harzianum* was also able to alter the expression of transcription factor *NAC1* and dehydrin *TAS14* genes, leading to cold tolerance in tomato plants (Ghorbanpour et al., 2018). *A. niger* enhanced heat tolerance of soybean and sunflower by control of ROS and peroxidative damage. This organism also increased plant growth and enhanced chlorophyll levels (Ismail et al., 2020).

#### Flooding Stress

During complete submergence, plants experience a strong decline in their photosynthesis rate and carbon availability. This is due to a lack of light or a reduced rate of CO_2_ diffusion, and to impaired respiration through reduced O_2_ availability (Perata et al., 2011). To survive in such conditions, microorganisms come up with a variety of physiological, biochemical, and molecular mechanisms to protect plants from this adverse condition.

Beneficial microorganisms induce plant growth and counteract flooding stress through production of ACC deaminase, discussed in section “salt stress.” Under flooded conditions, plant roots become hypoxic. In response to oxygen-limitation, the enzyme ACC synthase is synthesized in roots. Because there is limited oxygen, ACC cannot be converted into ethylene. Subsequently, the unused ACC is transferred to the shoots where oxygen is available, and ACC can be converted into ethylene. However, the overproduction of ethylene by plants results in their wilting, necrosis and chlorosis.

One way that beneficial microorganisms contribute to mitigate flooding-stress conditions is by their producing ACC deaminase that can convert ACC into *α*-ketobutyrate and ammonia, thus reducing the levels of ethylene (Tewari and Arora, 2016). In addition, beneficial microorganisms such as *Klebsiella variicola* was reported to mitigate the effect of flooding stress in plants by increasing plants’ adventitious roots. An increase in adventitious root growth encourages plant survival when the level of oxygen in the root zone is decreased (Kim et al., 2017). Maintaining proper shoot and root development especially under flooding stress is very essential for sustainable growth and development of plants. This is just one of many well-documented mechanisms by which the symbiotic relationship between plants and microorganisms works to the advantage of both.

#### Alleviation of Environmental Pollutants

One other stress that is alleviated by microorganisms is heavy metal stress. *Staphlococcus arlettae* alleviated chromate toxicity to sunflowers by suppressing the uptake and reducing the hexavalent form to the less toxic trivalent form (Qadir et al., 2020). Beneficial effects do not always occur. In commercial plantings of poinsettia, a report was received that said that in the presence of *T. afroharzium* T22, all the plants died, while in its absence, plants were healthy. It was discovered that the medium in which the plants were grown was sewage sludge with high levels of chromium. *T. afroharzianum* increased uptake of the element to toxic levels (observations of the first author).

The ability of a gibberellin-producing endophytic strain of *Penicillium janthinellum* in a tomato with tolerance to aluminum toxicity. Plant cell membranes were less damaged by application of the endophyte or exogenous gibberellic acid. Salicylic acid was upregulated by the presence of the fungus and It was concluded that either application of gibberellin or the endophyte counteracted the adverse effects of aluminum toxicity (Khan et al., 2015).

Amelioration of other environmental toxicants may be accomplished by the use of endophytic fungi. Cyanides are frequently present in mine tailings. Endophytic *T. afroharzianum* is able to take up and degrade metallocyanides. The polyphenols are toxicants produced in olive-processing systems and are toxic. If olive waste water is diluted and aerated, *Trichoderma* spp. degraded the polyphenols and appear to be a useful method to cleanse olive oil waste waters (Harman et al., 2004b).

Soils polluted by oily wastes are a serious problem. Researchers in Canada utilized *Pseudomonas* strains including *P. putida* which colonize plant roots. Treatment with the bacteria enabled plants to grow through enhancing stress resistance by mechanisms already described. This permitted roots to grow and provide a habitat for other microorganisms to proliferate. These other microflora degraded the hydrocarbons and thereby removed the pollution problem (Gurska et al., 2009). This system has been used commercially in Ontario. Research by the first author has been able to achieve this amelioration of oil pollution of soil by the use of *T. afroharzianum*.

#### Improvements in Internal Cellular Environments Allowing Efficient Functioning

An important component of higher-level functioning leading to EPHs is the maintenance of internal cellular processes critical for plant growth, development and induced resistance to both biotic abiotic stresses is the avoidance of damaging ROS by gene and protein upregulation of the pathways for alleviating and mitigating their damaging effects (see the preceding divisions of this section). This is a feature of many endophytic microorganisms discussed in this paper. Earlier in this review, we discussed the damage that ROS cause to cellular functioning, and these are described in (Nath et al., 2016). Since the mid-90s, many microbes have been shown to protect plants from ROS. These include mycorrhizae (Mo et al., 2016), *P. indica* (Sherameti et al., 2008), *Trichoderma* (Sherameti et al., 2008; Mastouri et al., 2012), and *Azospirillum* and *Rhizobium* (Fukami et al., 2018). In all of these cases, plants under stress were protected from damage against ROS. A very strong evidence of the capability of *Trichoderma afroharzianum* was provided by experiments in which tomato seedlings were exposed to the herbicide Paraquat™, which kills plants by inducing very strong ROS production to lethal levels. Inoculation of the seedlings overcame this damage (Mastouri et al., 2012). Alleviation of ROS damage in plants is accompanied by overexpression of the genes and proteins involved in managing ROS stress and was described in the earlier divisions of this section.

Thus, these endophytes are able to create an internal environment that maintains a cellular environment that is conducive to efficient operation of photosynthesis and all the other metabolic processes necessary for plant growth and amelioration of stresses. We have coined the term Optimized Internal Redox Environment (OIRE; Harman and Uphoff, 2019). This is a very important component of the beneficial effects of these beneficial organisms.

#### Improvements in Photosynthesis

The previous section described maintenance of cellular functions, including photosynthesis. However, photosynthesis is actually increased and improved by endophytes, which contribute to formation of EPHs. Within the genus *Trichoderma*, the presence of endophytes increases levels of photosynthetic pigments, which increase levels of proteins critical to photosynthesis (De Palma et al., 2016; Vitti et al., 2016; Pelhivan et al., 2017; Fu et al., 2018). Such changes are not restricted to *Trichoderma; Rhizobium* also demonstrates such effects (Chi et al., 2005). These beneficial effects are documented more fully in a paper devoted to this topic (Harman et al., 2019). Enhancement of photosynthesis is a very important attribute of endophytes that increase plant growth and limit adverse effects of biotic and abiotic stresses and that contribute to the formation of EPHs.

## Agricultural and Societal Benefits

### Meeting Food Demand and Reducing World Hunger

The global population is increasing rapidly and is predicted to be roughly 9 billion by the middle of this century. Concurrently, global agriculture is facing increasing competition for land, water, and energy in food production (Godfray et al., 2010). At the same time, the agricultural sector must cope with increasing temperatures, more erratic rainfall, more frequent droughts, rising sea-levels due to melting snow, and pest and pathogen pressure in crops around the globe (Wheeler and Braun, 2013). Importantly, despite a doubling of the global population in the past half century, the extent of global hunger has been reduced (Godfray et al., 2010). This was mainly due to contributions from the Green Revolution which increased crop productivity by the introduction of high yielding varieties, new irrigation schemes, and a broad range of agrochemical inputs to supply nutrients and manage pests, weeds, and pathogens. However, negative impacts of the Green Revolution started coming to the surface at the latter decades of 20th century in terms of soil degradation, environmental imbalance, and the emergence of chemical-resistant biotypes in pests, pathogens, and weeds.

In any case, global food requirements in the 2050s are expected to be double those of 2005. Unfortunately, annual productivity gains have been decelerating over the past two decades, and crop production area has been decreasing due to industrialization, desertification, and urbanization, which further constrains future food production. Therefore, there is urgent need for new initiatives to mitigate these challenges to the global food supply. Much evidence shows that careful selection and augmentation of efficient soil microbial strains is a potentially sustainable solution for raising agricultural production and reducing hunger globally. As mentioned previously, soil microbes play vital roles in helping crops to cope with multiple stresses induced by climate change such as higher temperature, more flooding and drought, and containing pests and pathogens. Soil microbes can also help to rehabilitate degraded soils by improving soil nutrient availability, suppressing soil pathogens, cleansing the soil of pollution and heavy metal contaminants, and preventing soil erosion.

### Environmental Benefits

Long-term monoculture production systems are one of the major factors contributing to soil degradation and contamination with inorganic pesticides, chemicals, and herbicide. Depending upon their concentration, soil contaminants can have destructive consequences on soil ecosystems, on the abundance and diversities of soil microbial communities, and on soil health. Soil microorganisms are not only crucial in crop production and soil health, but are also becoming important tools for cleaning up environmental pollutants (Morris et al., 2011). The process is known as bioremediation, where microorganisms are enlisted for their ability to degrade harmful chemicals and heavy metal contaminants in the soil by changing them into less harmful materials. Bioremediation using soil microbes is achieved by augmenting populations of selected microbial strains with high degradation abilities at the site of contaminant.

Several beneficial microbes have been reported to have high bioremediation potential for chemical pesticides, petroleum hydrocarbons, and heavy metal contaminants in the soil. Microbial services for soil rehabilitation are less costly, easier to employ, and more environmentally friendly than other methods now used such as the “muck, suck, and truck” approach. Bacterial and fungi species employed for bioremediation include *Trichoderma* species which are capable of degrading a wide range of polycyclic aromatic hydrocarbons (PAHs) such as naphthalene, phenanthrene, chrysene, pyrene, and benzene (Zafra and Cortés-Espinosa, 2015; Malla et al., 2018).

### Alleviation of Greenhouse Gases

It is well-established that microorganisms play critical roles in determining the concentrations of greenhouse gases such as carbon dioxide (CO_2_), methane (CH_4_), and nitrous oxide (N_2_O) in the atmosphere (Singh et al., 2010). Furthermore, use of chemical fertilizers in agriculture increases the greenhouse gas emission in three ways: (i) a huge amount of fossil fuels are utilized in the production of chemical fertilizers which is responsible for the emission of GHGs; (ii) fossil fuels are also used for the transportation and application of fertilizers and pesticides; and (iii) chemical fertilizers provide the substrates for methanogenesis and denitrifying bacteria which are responsible for the emission of methane, carbon dioxide, and nitrous oxide from soil into the atmosphere.

On the other hand, the application of beneficial microbes as biofertilizers, bio-stimulants, or biocontrol agents reduces the need for agrochemicals including fertilizers. Also, it reduces the habitat and sustenance for methanogens and denitrifying bacteria by competition for space, nutrition, and ecological niches in the soil, thereby reducing microbial sources of GHG emissions. Furthermore, if beneficial microbes such as *Trichoderma* are combined with conservation agriculture practices such as minimum tillage and with SRI as discussed in the next section, emission of GHGs can be reduced at the same time agricultural production is enhanced (Hawken, 2017).

For example, minimum tillage operations favor microbial communities dominated by fungi which decrease the microbial decomposition and respiration induced by soil disturbance during plowing. It has been reported that this could lead to sequestration of as much as 55 Pg organic carbon in the surface soil (Conant et al., 2003; Singh et al., 2010). SRI promotes alternate drying and wetting practice in paddy irrigation instead of continuous flooding, which is the usual practice in conventional rice farming. Continuous flooding supports the methanogenic activity of bacteria which emit large amounts of methane gas into the atmosphere. Flooded paddies are major contributors to GHG emissions from agriculture which account for around 11% of global anthropogenic methane emission (Stocker et al., 2013).

In China, a microbial preparation used with tea plants in their cultivation reduced emissions of N_2_O by 33.1–71.8%, while yield was increased by 16.2–62.2% relative to standard use of synthetic N fertilizers. These indicated once again that microbial enhancement of crop holobionts can reduce GHG emission from agriculture with favorable impact for less global warming with a gain rather than a sacrifice in production.

## Management and Delivery Systems for EPHs

The previous sections review the potential of various microorganisms to improve plant productivity. However, results may be inconsistent and suboptimal unless the organisms are properly used. This includes appropriate means of delivery to maximize and ensure that the organisms are able to colonize, protect and enhance plant growth and development. In addition to delivery systems, the way that microbial formulations are assembled and delivered can make a large difference in whether or not the organisms are able to provide the benefits anticipated. These are formidable challenges for their successful use.

### Delivery

The major challenges to be dealt with in the commercialization of microbial agents for improving crop production are inconsistent efficacy of the biological material, having a delivery system that can maintain the viability of the material, and ensuring appropriate physiological conditions of the microbial inoculants during transportation and handling (Arora et al., 2016). For the success of any microbial-based products in agriculture, selection of suitable delivery systems and formulation processes are equally important as the selection of effective microbial strain(s) (Zayed, 2016).

**Figure 3:**
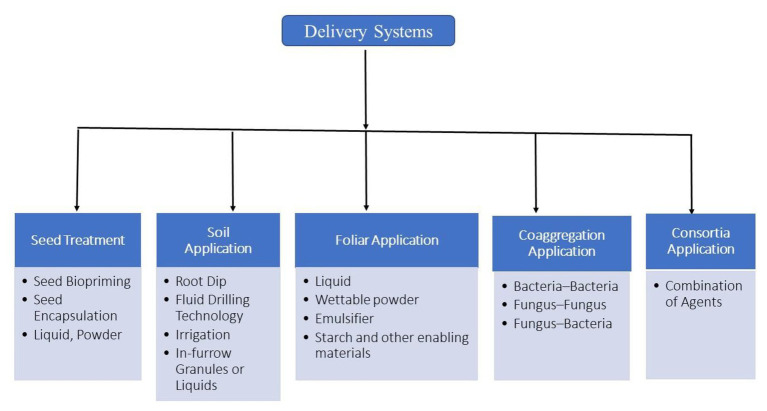
A diagram of different delivery systems employed in agriculture.

Based on considerations of survivability, mode of action, and local climatic conditions, there are several types of delivery systems for microbial inoculants. These can be directly applied to seeds by seed inoculation, to the roots by root dip methods, by drenching of young plants, to the plant and soil through drip irrigation or flooding, or to growing plants by foliar spray (Figure 3; Gašić and Tanović, 2013).

The basic characteristics of an effective delivery system include easy mode of delivery; enhancement of target activities with effective concentration; little or no ecotoxicity; time controlled release; solubility, stability, and effectiveness (Vejan et al., 2019). In the last several decades, exogenous application to plants, seeds or soil with microbial products has mostly been in the forms of powder, granules, slurry, or liquid. The dispersal system should provide the nutrients necessary for the microbial strains and protect them from desiccation, supporting release of the microbial cells as soon as this is required (Schoebitz et al., 2013).

Previously, the peat, clay, and liquid-based delivery systems were well-established; however, peat and clay-based carriers have proven difficult to sterilize, and they have a high chance of external contamination, plus low stability and viability during storage and handling. Similarly, liquid-based formulations may not be sufficiently protective to bacterial products during storage, transportation, and application into the soil. Stable liquid formulations are easier to achieve with spore-formers such as fungal spores and with gram-positive bacteria that form endospores; they are more difficult with gram-negative bacteria that lack resistant spore types.

Commercial practice usually requires that preparations have at least a year of shelf life without refrigeration or other special handling. Furthermore, suspension-based inoculations have several other demerits, such as settling of the microbial inoculants, blockage of spray nozzles during the application, and abiotic stresses affecting the viability of the spores (Bateman et al., 2007). It is essential to prepare formulations that meet the criteria of shelf life and ease of use if microbial formulations are to be accepted in large-scale commercial agriculture.

Recent advances in science have focused on developing various encapsulation technologies to deliver the microbial products more effectively and cheaply in the farming system. The processes of lengthening the cell viability of microbes by entrapping or coating them within polymeric materials that are permeable to nutrients, gases and metabolites is known as encapsulation. Encapsulation has been divided into two categories based on the bead size: (i) macro encapsulation (a few mm to cm), and (ii) microencapsulation (1–1,000 μm; John et al., 2011).

Synthetic polymer, biopolymer, and different kinds of organic/inorganic materials are used in encapsulating microbial cells for agricultural use. The three major benefits related to encapsulation technology compared to the traditional delivery system include: (i) enhanced efficacy due to increased surface area; (ii) enhanced microbial activity due to higher penetration efficiency; and (iii) higher dispersion on plant surfaces due to smaller particle size (Sasson et al., 2007). Encapsulation may also increase stability and shelf life and may be essential if non-spore forming organisms are used (Harman and Custis, 2006).

Bioformulation improves the performance of microorganisms by preserving them and delivering them to their targets more reliably. (Stephens and Rask, 2000) proposed that good microbial carriers are characterized by their effectiveness in supplying the right number of viable cells in excellent physiological conditions, and at an appropriate stage or time. The carrier materials that increase the storability of the products generally include fine clay, peat, talc, lignite, alginate, polyacrylamide beads, starch, diatomaceous earth, vermiculite, cellulose (carboxymethyl cellulose), and polymers, especially xanthan gum (Digat, 1991; Harman and Custis, 2006). Furthermore, in various local conditions, charcoal, farmyard manure, vermicompost, compost, bagasse, press mud, and sterilized grains such as rice, corn, millet and barley have also been successfully used as carriers for different kinds of microorganisms (Connick et al., 1991; Schisler et al., 2004; Pitt and Hocking, 2006; Dorner, 2009; Harman et al., 2010).

Microbial inoculants can be formulated as dust, seed dressing powder, micro-granules, water dispersal, wettable powder, emulsifiers, suspension concentrates, oil dispersion, emulsions, capsule suspension, and ultra-volume formulations (Knowles, 2006). Granular formulations protect microorganisms from ultraviolet light. A recently-developed soybean oil-based formulation increases the half-life of the conidia and the performance of *T. asperellum*. The oil-based formulation has more benefits than a water-based formulation because it remains in contact with the plant surface for a longer time, evaporates much less, and can also be applied as an emulsion (Batta, 2007).

Some researchers (Xue, 2002) have expressed concerns regarding inconsistent results from the use of single strains and have proposed using multiple strains or consortia of microbes, so there has been interest among researchers in using multiple species or strains of beneficial microbes in aggregation or in consortium form to exploit the potential synergy between them. Bioformulation based on consortia of beneficial microbes should have more impact in farming systems due to utilization of all possible mechanisms of action inherent in the different strains within the consortium. Application of combinations of strains resulted in higher levels of disease suppression compared to applying individual strains (Meyer and Roberts, 2002; Roberts et al., 2005).

In the senior author’s experience, single strains or at most three strains can be effective if the strains are carefully selected, if the formulations are appropriate, and if they are used with appropriate management systems. Limiting the number of strains is essential if registration is required, since most registration requirements include toxicology evaluations that are very time-consuming and expensive. Full registration packets typically require several tens of thousand dollars for each strain, and if multiple strains are used the total cost can exceed a million dollars. Regardless of the strain(s), delivery system, or formulation used, it is essential that any product or strategy be tested rigorously to ensure reliable results in the field.

### Application of Exogenous Inoculum

Obviously, the selection of effective microbial strains is critical in bio-based product development. These strains should be sufficiently competitive with indigenous soil microbial populations to survive, as well as being compatible with other inoculants and native microorganisms along with their colonization efficiency in plant rhizoplane (Berendsen et al., 2012). It is well-established that exogenous inoculation of beneficial microorganisms in soil increases plant growth, promotes plant nutrient uptake, enhances plants’ tolerance to biotic and abiotic stresses, and suppresses plant diseases (Roiger and Jeffers, 1991). Due to the dynamic and self-sustaining propensities of microorganisms, many strains or species can establish themselves well in the soil so that there would not be a need for repeated applications (Lucas et al., 1990).

As mentioned already, microbial species and strains have differential potentialities to serve the plant communities within and with which they reside, utilizing different mechanisms and colonizing different niches in the rhizosphere and rhizoplane regions. Many will inhabit the plants’ endospheres. The carefully-selected application of microbial strains can fill vacant niches in the soil and establish communication with plants and other strains differently than do the indigenous microbes that are already present in the soil. Some microbial strains can vigorously thrive in more variable environments than can others. These can quickly capture spaces and nutrients in the plant rhizosphere, making these resources unavailable to potential pathogenic invaders.

The effects from exogenous application of plant microbes are not always consistent across fields and crop species. However, research has been undertaken focusing on how to prepare and adapt the rhizosphere environment for colonization of specific exogenously-applied microbial strains by rhizosphere engineering (Ryan et al., 2009), and on how to deliver them more effectively for establishment. Plant microbial colonization is also greatly affected by indigenous microbes, by the soil’s physiochemical environment, and by plant species and even genotypes within the crop species. Thus, the application of beneficial microbes requires an understanding of which microbial strains are more suited for which soil quality, which farming system, or even which crop genotypes, and consideration of which plant species and genotype-specific strains might respond more favorably to inoculation (Siddiqui and Shaukat, 2003).

Host variation along with plant variety will affect a plant’s response to beneficial microorganisms (Smith and Goodman, 1999). These authors further elaborated that hosting beneficial microbes in any plant species is influenced by that plant’s genes. (Tucci et al., 2011) have reported differential rates of gene expression after the inoculation of *Trichoderma* strains in different varieties of tomato. Thus, understanding the roles of individual plant genotypes in supporting or impeding plant-microbe interactions is a necessary step in the success of any beneficial microbes.

### Mobilization of Existing Soil Populations

Agricultural soil is a vast reservoir of microbial biomass and diversity. It is estimated that 1 g of rhizosphere soil contains more than 100 species of microbes and 10^8^–10^11^ viable cells (Berendsen et al., 2012). Furthermore, plant rhizosphere and rhizoplane shelter different microbiomes with a diversity of microbial traits that is highly relevant to plant growth and health. The proper management and enhancement of these existing populations of soil microbes may provide excellent services for plant growth and help plants cope with multiple stresses, thereby reducing the need for use of agrochemicals (Saleem et al., 2019).

High tillage, the use of pesticides and chemical fertilizers, continuous monoculture, flood irrigation, reduced crop rotation, and lack of organic matter in the soil all negatively impact the soil microbial population and its diversity. On the other hand, cover cropping, crop rotation, intercropping, minimum tillage, system of rice intensification methods, the addition of organic manure, and organic amendments to the soil all support microbial and microbiome diversity and population. The addition of microbial probiotics, biochar, and organic manure with these different agronomic practices has been reported to increase microbial abundance and activity in the soil. Thus, it is possible to increase the efficacy of indigenous microbial populations by complementary crop and soil management practices and by purposeful enhancement of the soil biota (Pang et al., 2017; Meng et al., 2019).

## Enhancing Microbial Endowments with Changes in Crop and Soil Management

### The System of Rice Intensification

Rice is the staple food for more than 50% of the global population. A rice production system known as the system of rice intensification developed in Madagascar and validated in over 60 countries is currently gaining currency because of its higher productivity per seed grain, per drop of water, per unit area of land, and per unit cost of production. SRI relies on alternate wetting and drying (AWD) of rice paddies rather than on their continuous flooding, and it controls weeds by actively aerating the upper layer of soil around plants by use of a rotary weeder, rather than by using herbicides. It enhances soil nutrients by the addition of organic matter rather than with chemical fertilizer (Thakur et al., 2010; Thakur and Uphoff, 2017). AWD by itself boosts water use efficiency in rice production (Carrijo et al., 2017). The set of recommended practices raise factor productivity in addition to lowering water requirements (Stoop et al., 2002).

These SRI effects have been reported in over 1,000 papers and are already being achieved by around 20 million farmers using its methods in Asia, Africa and Latin America (SRI-Rice, n.d.). The productivity gains are achieved not by making changes in crop genetic potential and applying external agrochemical inputs but by making synergistic changes in soil, crop, and water management practices to create a more favorable environment for beneficial microbes such as mycorrhizal fungi and phosphorus-solubilizing microbes.

Continuous flooding has deleterious effect on rice plant physiology and anatomy as well as suppression of aerobic soil microbes. For example, flooding has deleterious effects on rice root systems by deforming cells in their cortex to create aerenchyma (air pockets) which affect the transport of water and nutrients (Kirk and Bouldin, 1991). Under flooded soil conditions, lower metabolism and ion transport reduces the growth of rice roots and canopy (Barison and Uphoff, 2011). Further, depletion of oxygen in the bulk soil of flooded rice paddies enhances methanogenic archaea rather than aerobic plant growth-promoting microorganisms (Liesack et al., 2000) and methanotrophs, that consume methane produced by methanogens (Rajkishore et al., 2013).

The effects of inoculating SRI-grown rice plants with *Trichoderma* have been evaluated in Nepal to assess possible synergy between SRI’s crop management methods and enhancement of the plants with beneficial endophytes, thereby creating EPHs (Khadka and Uphoff, 2019). Trials were conducted in Nepal with two rice varieties, one improved and the other local. It was found with two seasons of trials that there was a 75% average increase in grain yield from the two varieties when rice seedlings were treated with a native isolate of *T. asperellum* before they were transplanted and raised in a field managed organically according to SRI-recommended practices, compared with farmers’ usual practices, reduced or including inorganic fertilization. There was a 58% average increase in yield from these varieties managed with SRI methods but with no microbial inoculation; and the increase from plants inoculated with *Trichoderma* but managed otherwise with conventional methods was 67%. The latter methods included higher plant density, flooding of fields, and inorganic fertilization. These results were not surprising since growing rice under aerobic soil conditions is more favorable for microbes such as *Trichoderma* than is hypoxic flooded soil.

There was a 58% average increase in yield from these varieties when they were managed with SRI methods but no microbial inoculation. However, yield was further increased by inoculation with *Trichoderma*. Among all the replicated trials, the yield was highest (6.5 t ha^−1^) when inoculation was combined with SRI methods (more aerobic soil, more organic matter). These results should not be surprising since SRI soil conditions are more favorable for aerobic microbes such as *Trichoderma* than is hypoxic flooded soil with no enhancement of soil organic matter.

Research in Malaysia found similar results with a significant increase in seedling growth, germination rate, seed vigor index, and leaf chlorophyll content when rice plants grown under SRI management were inoculated with *T. asperellum* sp. SL2 compared to their cultivation with conventional methods plus inoculation with *T. asperellum* (Doni et al., 2017, 2019b). In further research, it was determined that there was greater net photosynthesis, more internal CO_2_ concentration, greater water use efficiency, plant height, tiller number, root length, and fresh root weight in rice plants that were inoculated with *T. asperellum* compared to uninoculated plants (Doni et al., 2019b). This research confirmed that EPH effects can be heightened by the use of conducive crop management practices.

### No-Till With Cover Crops and Other Management Practices

There is growing interest in alternative cropping methods not only to reduce farmers’ costs of production but also to increase their yields and reduce adverse environmental impacts of farming, particularly soil erosion and agrochemical contamination of soil and water. What is known as conservation agriculture combines reduced mechanical tillage such as no-tillage or minimum tillage, plus crop residue retention on the soil, the use of cover crops, and crop rotation. It has been reported that about 13% of global croplands, 200 million ha in 80 countries, are covered by conservation tillage (Kassam et al., 2014).

Both cover crops and reduced tillage practices improve soil systems’ physical and chemical properties, which has a positive impact in the biological properties of soil. Reduced tillage improves the soil’s structure through better aggregation, soil organic matter, water infiltration, water-holding capacity, and less soil erosion. Cover crops cultivation increase nutrient inputs through crop residue decomposition, biological N fixation, and root exudates, both for succeeding crops and microbial communities (Schmidt et al., 2018). This enables cover crops to enhance microbial abundance and microbial diversity. Further, broader metabolic capacities of microbes have been reported in cover crop-cultivated fields compared to non-cover crop-cultivated fields that enhance the fungal populations in the soil (Schmidt et al., 2018).

Conservation tillage greatly influences the soil microclimate, dissemination and decomposition of crop residues, and nutrient recycling. All of these changes have positive effects in soil microbial diversity and abundance (Li et al., 2019). It has been reported that conservation tillage conditions the soil pH to make it more suitable for crop growth and microbial diversity and population, which also enhances the fungal hyphal networks in the soil. Higher antibiosis abilities of endophytic and root-zone bacteria, both within and around roots, have been reported with crop rotation and conservation tillage (Peterson et al., 1993). Furthermore, several reports are available that the population, diversity and root colonization rates of arbuscular mycorrhizal fungi (AMF) were consistently reduced by the tillage operation in soil (Säle et al., 2015; Jesus et al., 2016; Schmidt et al., 2018) Enhancement of this symbiotic fungus in crop plant roots is a good strategy for enhancing crop supply of nutrients such as phosphorus and for giving the plants more resilience to drought stress.

## Summary

This paper offers a comprehensive review of the knowledge and practice for using endophytic microorganisms to enhance and maintain more beneficial systems of crop production. Most of the cases reported involve endophytic bacteria and fungi symbiotically affecting the functioning of plants, but in some instances beneficial microorganisms’ principal means of benefitting agriculture and the environment are by other mechanisms such as competition and mycoparasitism.

The review considered first different patterns of endophytic root colonization (Figure 1). Then the next section dealt with mechanisms by which microbes have beneficial effects on crop plant performance. Figure 2 provided a synoptic view of the mechanisms that can be found in nature. Colonization of the interior of plants, particularly their roots, can induce systemic reactions in the whole plant that give it more resistance or tolerance to pests and pathogens, that enhance photosynthesis, or that improve plant functioning at the cellular level.

Beneficial endophytes produce SAMPs that interact with cell membranes and result in signal transduction *via* MAP kinases. This results in higher expression of many proteins and genes, including those that give rise to coordinated improvements in disease suppression and to increased resistance to abiotic stresses such as drought, salt, flooding, and adverse temperatures.

These systemic responses also improve and maintain photosynthesis. This together with improved nutrient efficiency and acquisition, including N_2_ fixation, provide the essential components for plant growth and development. Improving photosynthesis is critically important. Plants require the energy and fixed carbon compounds to support both the numerous changes involved is systemic resistance systems and to provide the wherewithal for plant growth and development.

Provision of beneficial endophytes by itself cannot provide total reliability and results. There need to be management practices and good delivery methods that ensure viability and efficacy of applications. The components of effective delivery systems include effective seed treatments or soil applications, and effective organisms that must be selected from either single strains or consortia of different strains since only a few strains or species can enter into mutually-beneficial relationships with plants (Figure 3). Management strategies that can enhance the abundance and diversity of beneficial microbial populations include conservation agriculture farming systems, System of Rice Intensification practices, and the management of native populations to strengthen the effectiveness of soil microbial populations. No-till and minimum-till systems can be part of soil management and are particularly effective if accompanied by cover cropping or other methods of maintaining soil coverage with vegetation or other organic materials. There can, however, be disadvantages. For example, leaving corn residue on soil surfaces may provide a source of inoculum of pathogenic *Fusarium* for the next year’s crop. In addition, heavy plant residues may make till and cultivation difficult or impossible.

**Figure 4:**
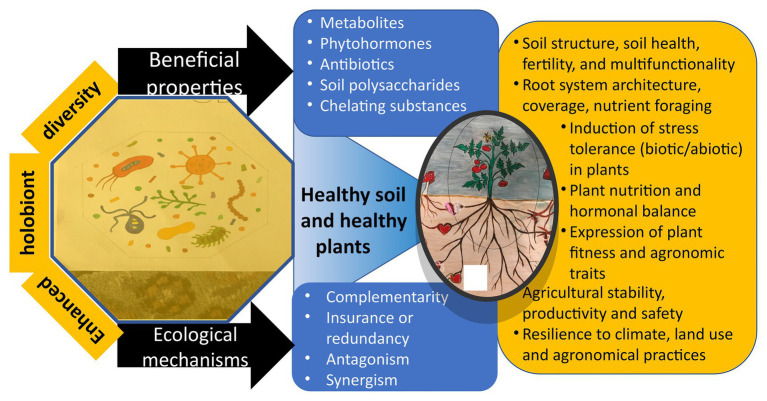
Summary of the various components and management systems that interact to provide optimal performance of plant agriculture.

These changes in crop and soil management improve soil health and increase the organic matter in soils (Figure 4). With these enhancements, the prospects for meeting the world’s food needs are greatly improved, along with the opportunities to avoid the worst consequences of climate change by abating greenhouse gas emissions. Given the great challenges and uncertainties that we presently face, there are no guarantees of success. But understanding and taking advantage of the potency of some of the world’s tiniest organisms to make our agriculture more sustainably productive could improve the future for farmers and consumers and provide some simple, low-cost tools for alleviating some of the largest and most basic problems confronting our society.

## Author Contributions

All authors listed have made a substantial, direct and intellectual contribution to the work and approved it for publication.

### Conflict of Interest

The authors declare that the research was conducted in the absence of any commercial or financial relationships that could be construed as a potential conflict of interest.
